# Supplementary material for: Therapeutic Targeting of Protein Disulfide Isomerase PDIA1 in Multiple Myeloma
Source: Cancers (Basel). 2021 May 28;13(11):2649. doi: 10.3390/cancers13112649 (PMC8198550; doi:10.3390/cancers13112649)
Supplement: Supplementary file 1 [file cancers-13-02649-s001.zip › Cancers_supp_file/Cancers_supp_file_5.26.21.pdf]

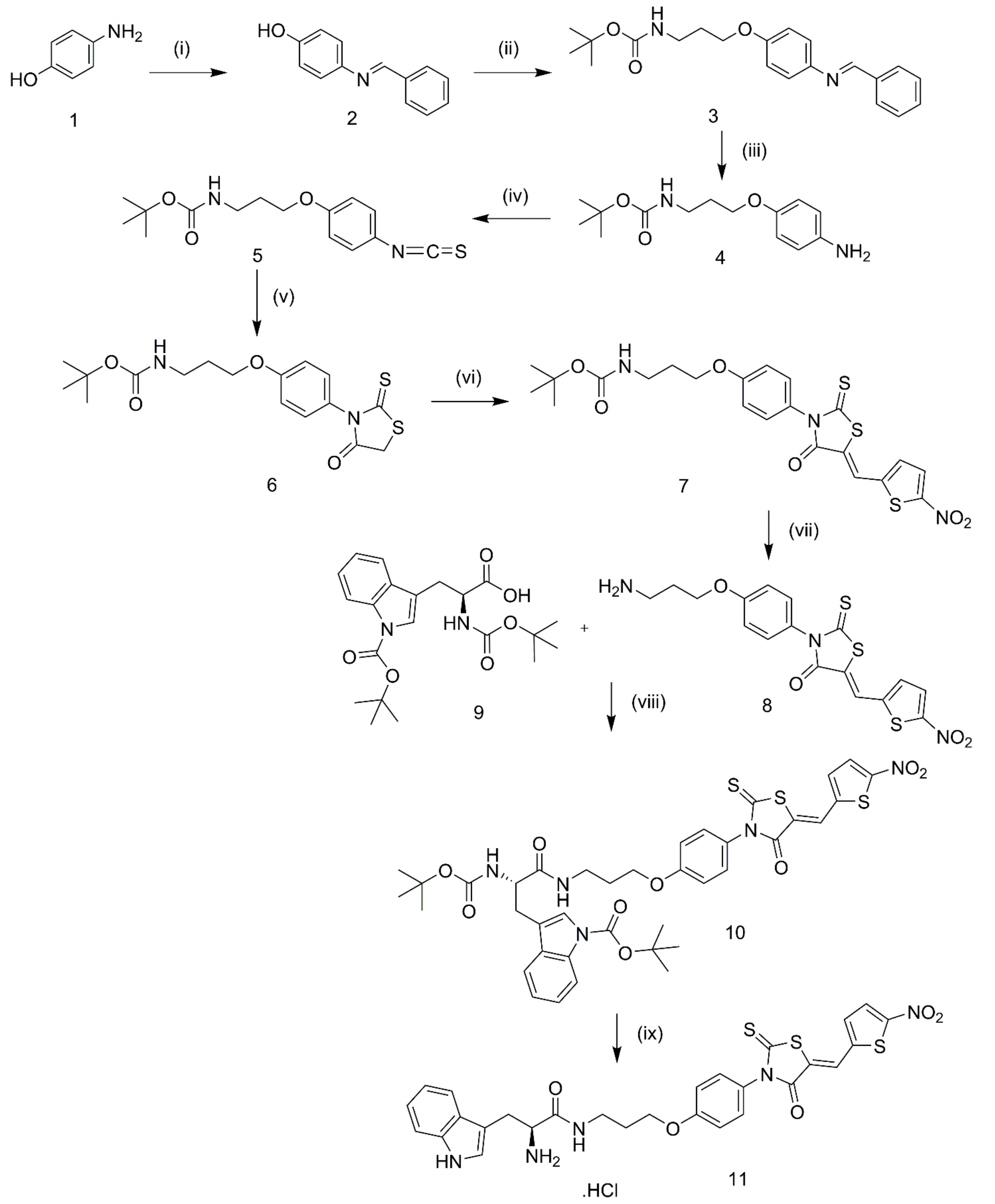

Reagent and conditions: (i) PhCHO, trimethyl orthoformate; (ii) Cs_2,CO_3, DMF, Boc-NHC_3H_6Br, 20°C, 4 h; (iii) $Pd(OH)_2$, NH_4COOH, Ethanol; (iv) $C(S)Cl_2$; (v) Et_3N, $MeOC(O)CH_2SH$; (vi) NaOAc, AcOH, 5-nitrothiophene-2-carbaldehyde, 90° C; (vii) 4N HCl in Dioxane, 20° C, 4 h; (viii) DIPEA, DMF, HATU 20° C, 4 h; (ix) 4N HCl in Dioxane, 20° C, 4 h

Supplemental Figure 1A. Chemical synthesis scheme of CCF642 analogues. The reaction conditions and details are described in medicinal chemistry method section.

B.

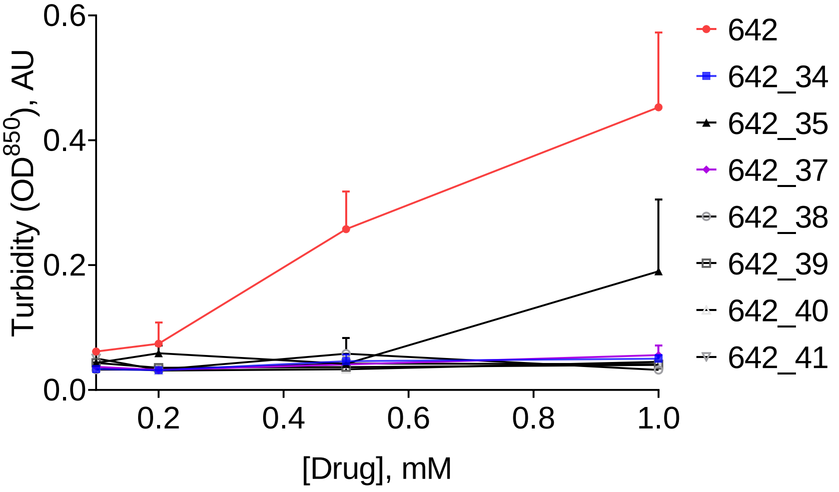

Table 1.

Drug Name	IC50, µM	Chemical Structure	ClogP	LogS
642_34	0.12	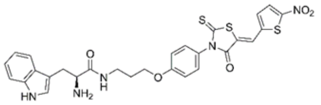	4.056	-7.208
642_35	0.15	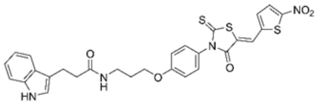	4.953	-7.531
642_37	0.18	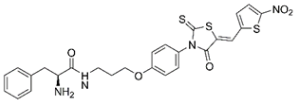	4.066	-6.461
642_38	0.32	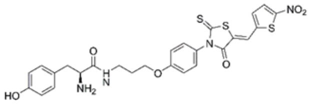	3.399	-6.299
642_39	8.31	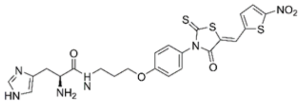	1.895	-5.48
642_40	3.06	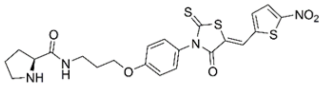	3.3526	-5.295
642_41	0.39	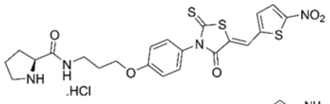	3.3526	-5.795
642-34-amine	N/D	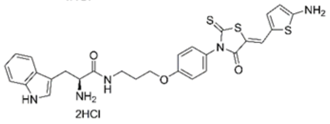	3.196	-6.802

Supplemental Figure 1B. Solubility analysis for CCF642 and its analogues. Turbidity of CCF642 and its analogues were determined by absorbance at OD^{850}. Briefly, 100 mM stock of each analogues in DMSO were directly diluted in water mixed briefly at room temperature and turbidity was determined by measuring optical density at 850 nm on multiplate reader Biotek Synergy plate reader. Each experiment was performed in triplicate and plotted in GraphPad Prism 8.0.

Supplemental Table 1. The physio-chemical properties of CCF642 and its analogues. The physio-chemical properties of CCF642 and its analogues along with IC50 against MM1.S cells are presented. Data is representative of three independent experiments. The IC50 was calculated in GraphPad Prism using nonlinear regression, dose-response inhibition.

C.

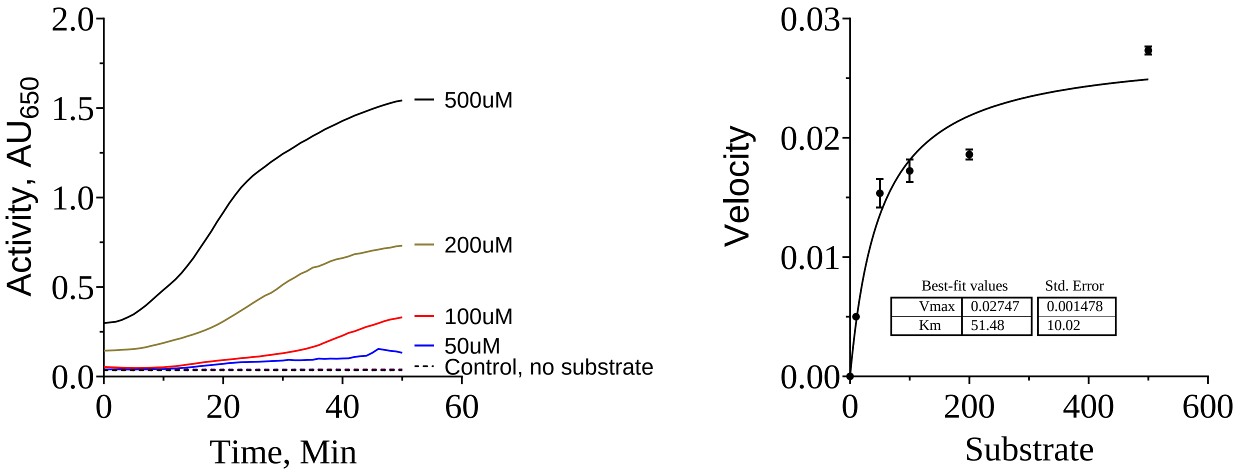

D.

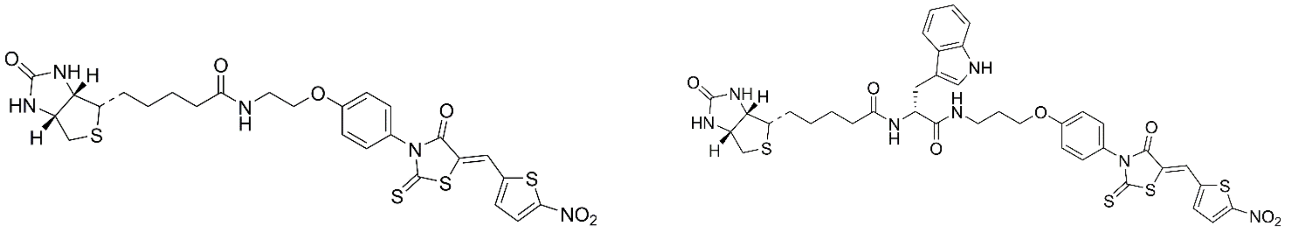

CCF642-Biotin

CCF642-34-Biotin

Supplementary Figure 1C. CCF642-34 is a potent PDIA1 inhibitor in insulin reduction assay. The reductase inhibitory activities CCF642-34 was determined by measuring the aggregation of insulin in the absence of accurately folded protein due to lack of correct disulfide bonds. Recombinant PDIA (1 μM) was incubated with varying concentrations of CCF642-34 for 1 hour followed by addition of insulin (100 μM). The insulin aggregation was monitored at 650 nm on Biotek Synergy plate reader.

Supplemental Figure 1D. Chemical structures of biotin labeled CCF642 and CCF642-34. CCF642-Biotin and CCF642-34-Biotin were used for drug target validation in MM1.S cells for probing the cell lysates with streptavidin in western blot analysis. PDIA1 was confirmed by the identity of the peptides in LCMS, raw data is deposited to the ProteomeXchange consortium via the PRIDE59 partner repository.

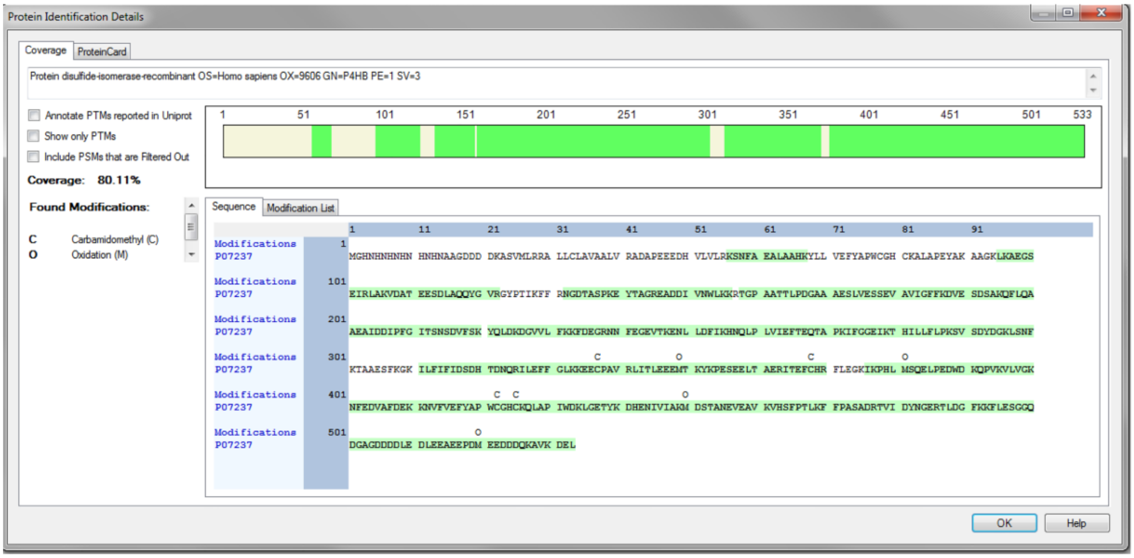

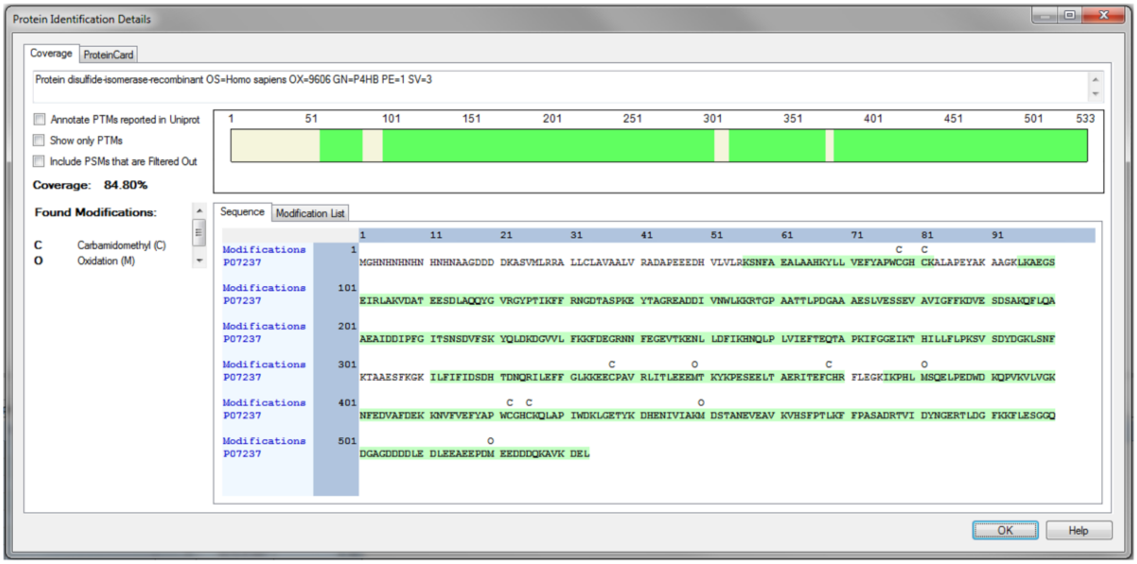

Supplemental Figure 1E. Sequence coverage of PDIA1 protein. Proteins pull down with CCF642-34-biotin were fractionated on 1D-SDS PAGE. On western blot there were two immune-positive bands; first band above 50Kda and second band at a lower molecular weight ~10Kda. Both band 1 and band 2 were cut from the gel, tryptic digested, and analyzed with LC MS/MS. Top panel: Band 1 was matched to PDIA1 with 80% coverage. Bottom Panel: Band 2 was matched to PDIA1 with 85% coverage.

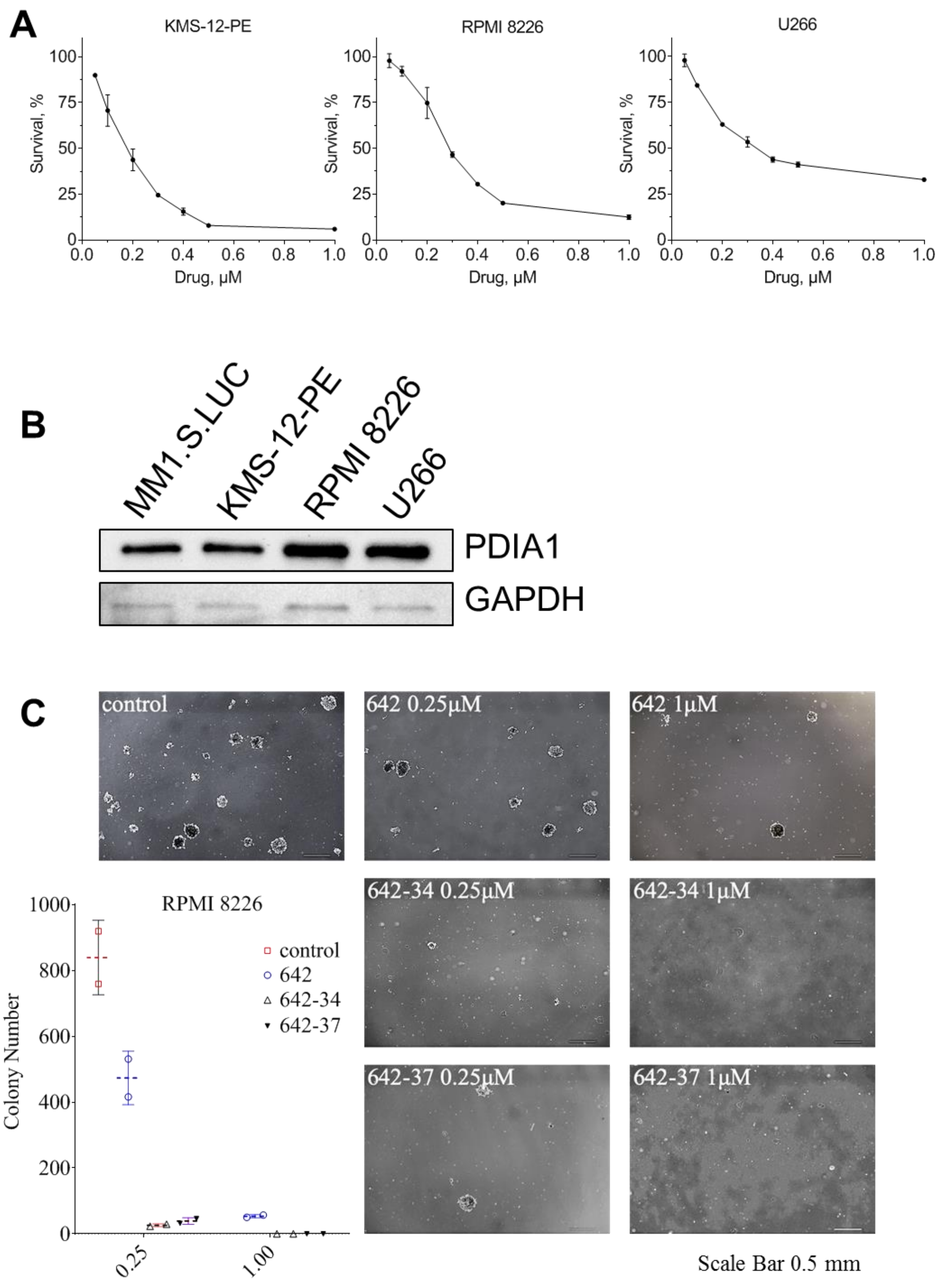

Supplemental Figure 2A. Toxicity of CCF642-34 on additional multiple myeloma cell lines. Toxicity of CCF642-34 was tested on additional multiple myeloma cell lines; KMS-12-PE, RPMI 8226, and U266. IC50 values were 0.165, 0.29, and 0.37 μM, respectively.

Supplemental Figure 2B. : **PDIA1 expression of multiple myeloma cell lines.** Multiple myeloma cell lines that are used in this study; MM1.S LUC., KMS-12-PE, RPMI 8226, and U266 were compared for PDIA1 expression in a western blot analysis. 10ug of total protein were loaded for each cell line and probed with PDIA and GAPDH antibodies.

Supplemental Figure 2C. The PDI inhibitors CCF642, CCF642-34, and CCF642-37 restrict the colony forming ability of MM cells. RPMI 8286 multiple myeloma cells were grown in semi-solid media (MethoCult™, H4435; STEMCELL Technologies) in a colony forming assay. Colony numbers were plotted for each treatment given at two indicated doses. Scale bar was drawn into scale (0.5 mm).

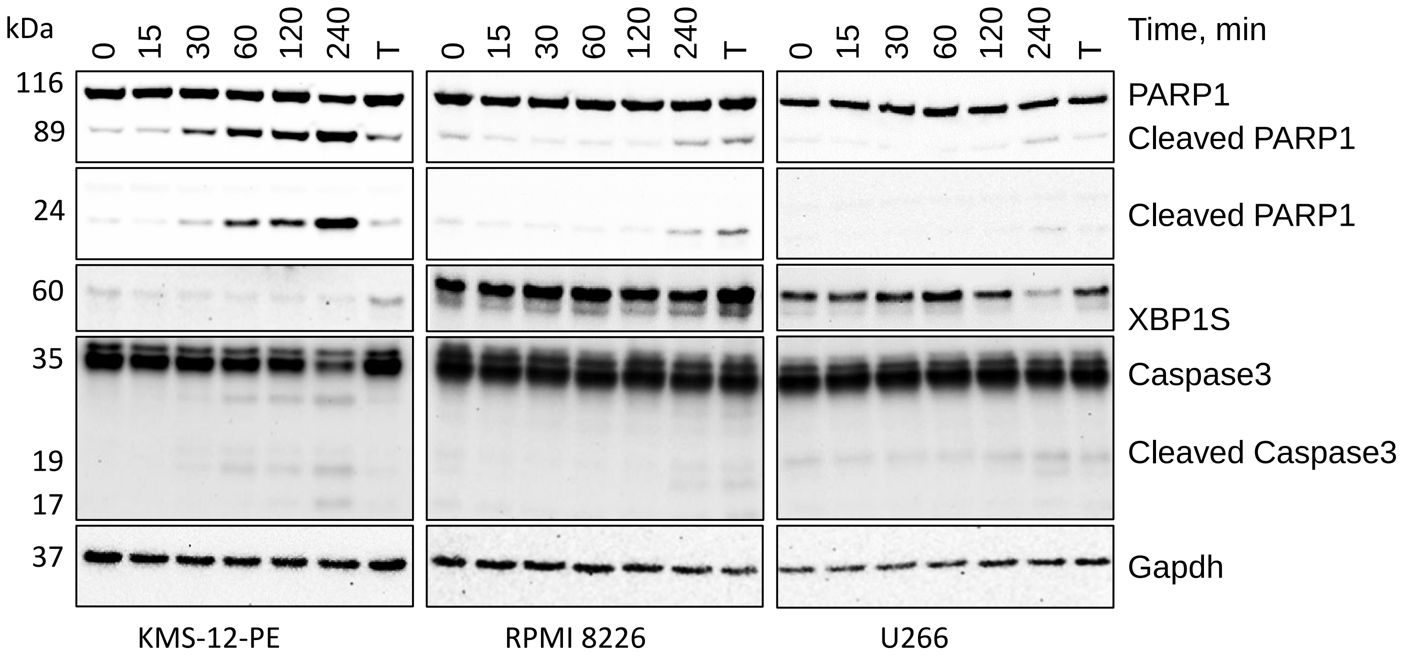

Supplemental Figure 2D. PDIA1 inhibition by CCF642-34 induces acute endoplasmic reticulum (ER) stress response and lead to apoptosis in MM cell lines. Multiple myeloma cell lines; KMS-12-PE, RPMI 8226, and U266 were treated with 3 μM of CCF642-34 for 15, 30, 60, 120, and 240 minutes. Tunicamycin (T) was used as a control. The status of ER sensor (XBP-1S), and apoptosis markers (cleaved Caspase3 and PRP1) were examined by western blot analysis.

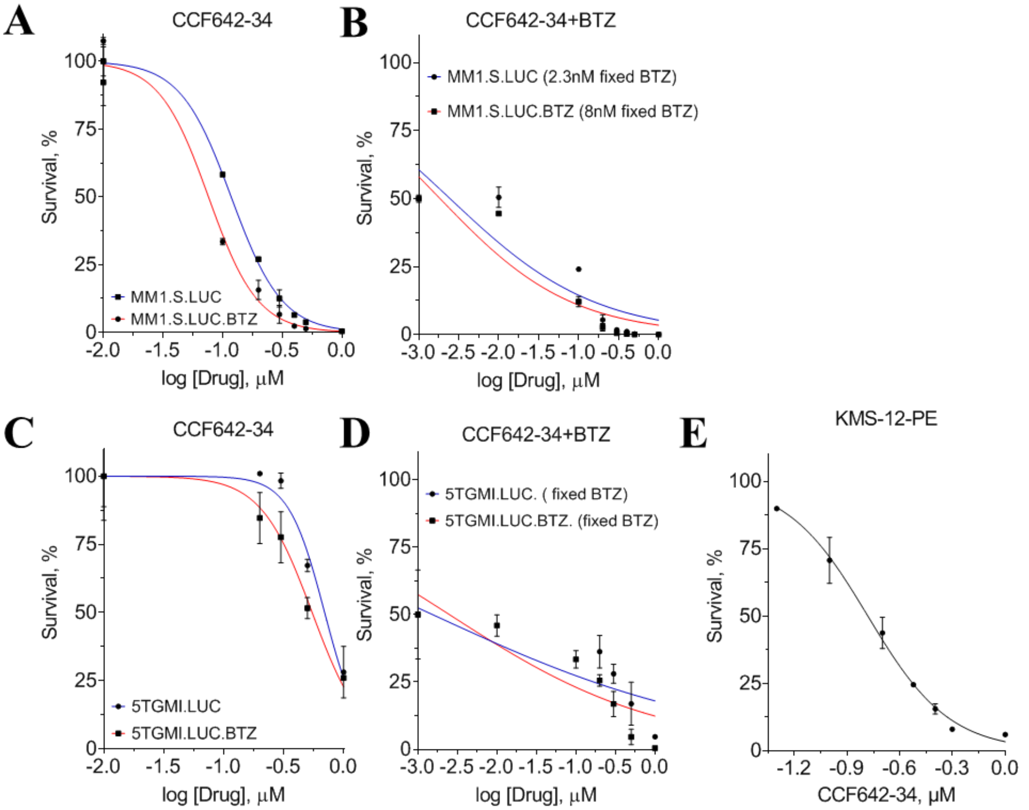

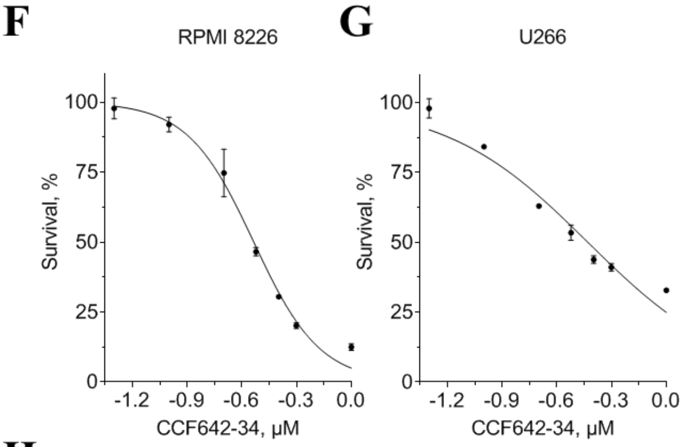

MM Cell Line	IC50, μM
MM1.S.LUC	0.118
MM1S.LUC.BTZ.res	0.07
5TGMI.LUC	0.694
5TGMI.LUC.BTZ	0.546
KMS-12-PE	0.165
RPMI 8226	0.291
U266	0.371

H

CCF642-34		
fa	Dose	log(fa/fu)
0.233	100	-0.5174394
0.265	150	-0.4430415
0.253	200	-0.4702001
0.279	250	-0.4123311
0.3275	300	-0.312481

BTZ		
fa	Dose	log(fa/fu)
0.4465	1	-0.093296162
0.467	1.5	-0.057410328
0.5315	2	0.054793674
0.57	2.5	0.1224064
0.628	3	0.227416704

CCF642+BTZ		
fa	Dose	log(fa/fu)
0.6005	101	0.17699623
0.6175	151.5	0.20800552
0.6435	202	0.25648902
0.6285	252.5	0.22834646
0.6895	303	0.34647267

X	Y
fa	CI
0.601	0.444
0.618	0.490
0.644	0.574
0.629	0.524
0.690	0.770

fa, fraction affected
CI, combination index

Supplemental Figure 3A-G. Nonlinear regression plots of cell viability assays. Cell viability were plotted in a nonlinear regression, dose-response inhibition graphs to calculate the IC50 of inhibitors for MM1.S.LUC, KMS-12-PE, RPMI 8226, and U266.

Supplemental Figure 3H. Synergistic drug combination assay for CCF642-34 and BTZ. MM1.S.LUC cell line was exposed to CCF642-34, BTZ, and CCF642 plus BTZ for 72 hrs for drug combination testing according to Chou and Talay[1] method. If fraction affected (fa) is zero, there is no effect on cells. If fa is 1, it means all cells are dead.

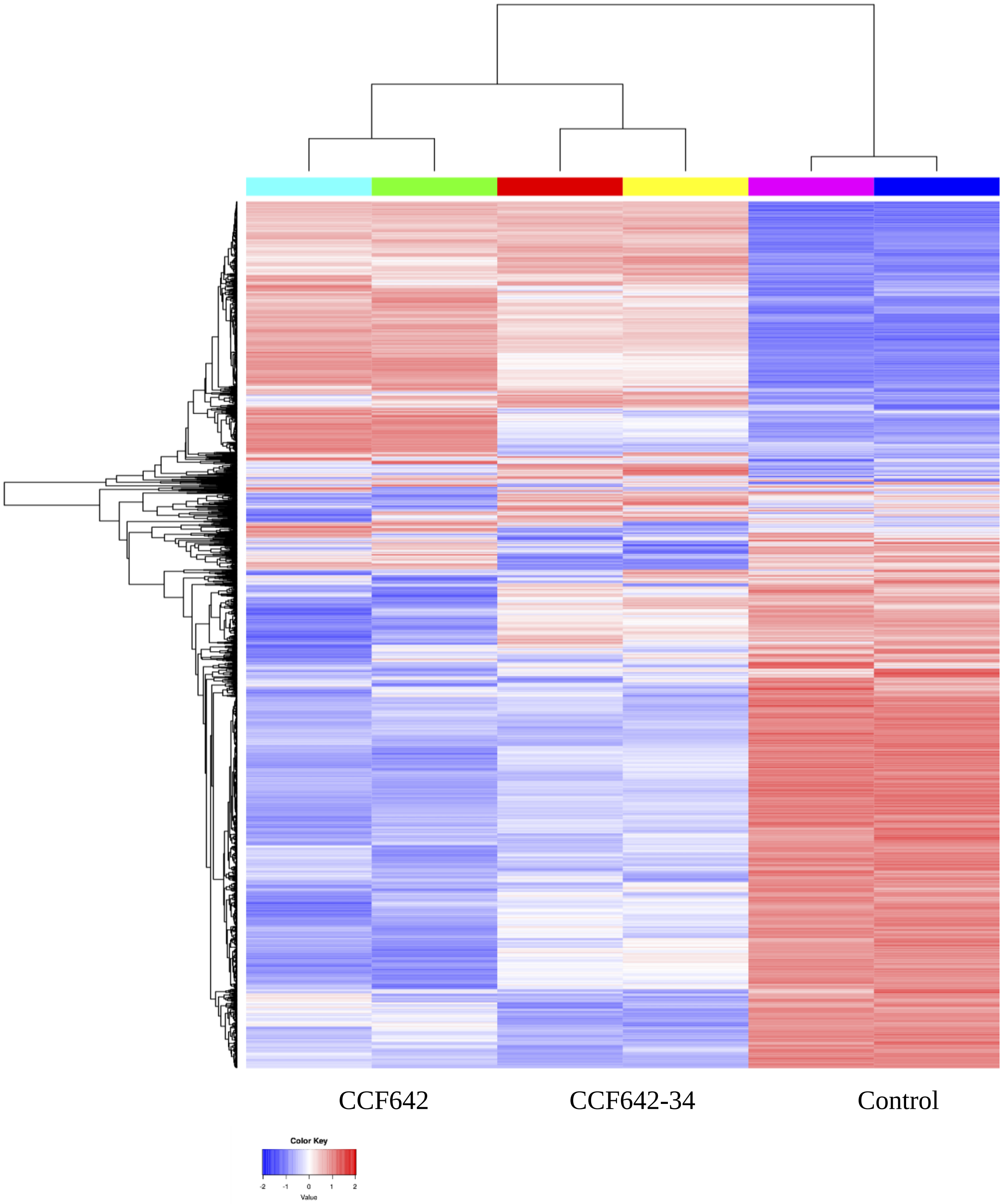

Supplemental Figure 4A. Gene set enrichment analysis of differential expression
A) Hierarchical clustering and heatmap of the gene expression in MM1.S cells treated with vehicle, CCF642-34, or CCF642, all genes were ranked by standard deviation across all samples and the top 5000 significantly expressed genes were used for unbiased hierarchical clustering using web-based server http://bioinformatics.sdstate.edu/idep/.

B.

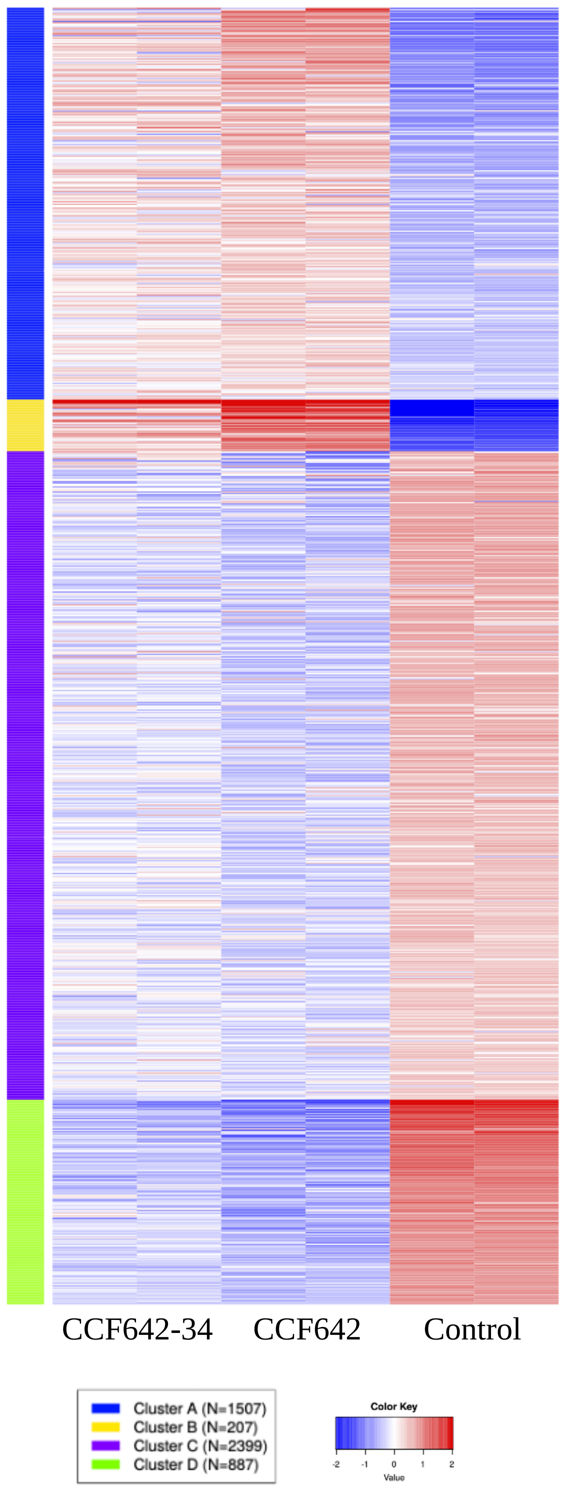

C.

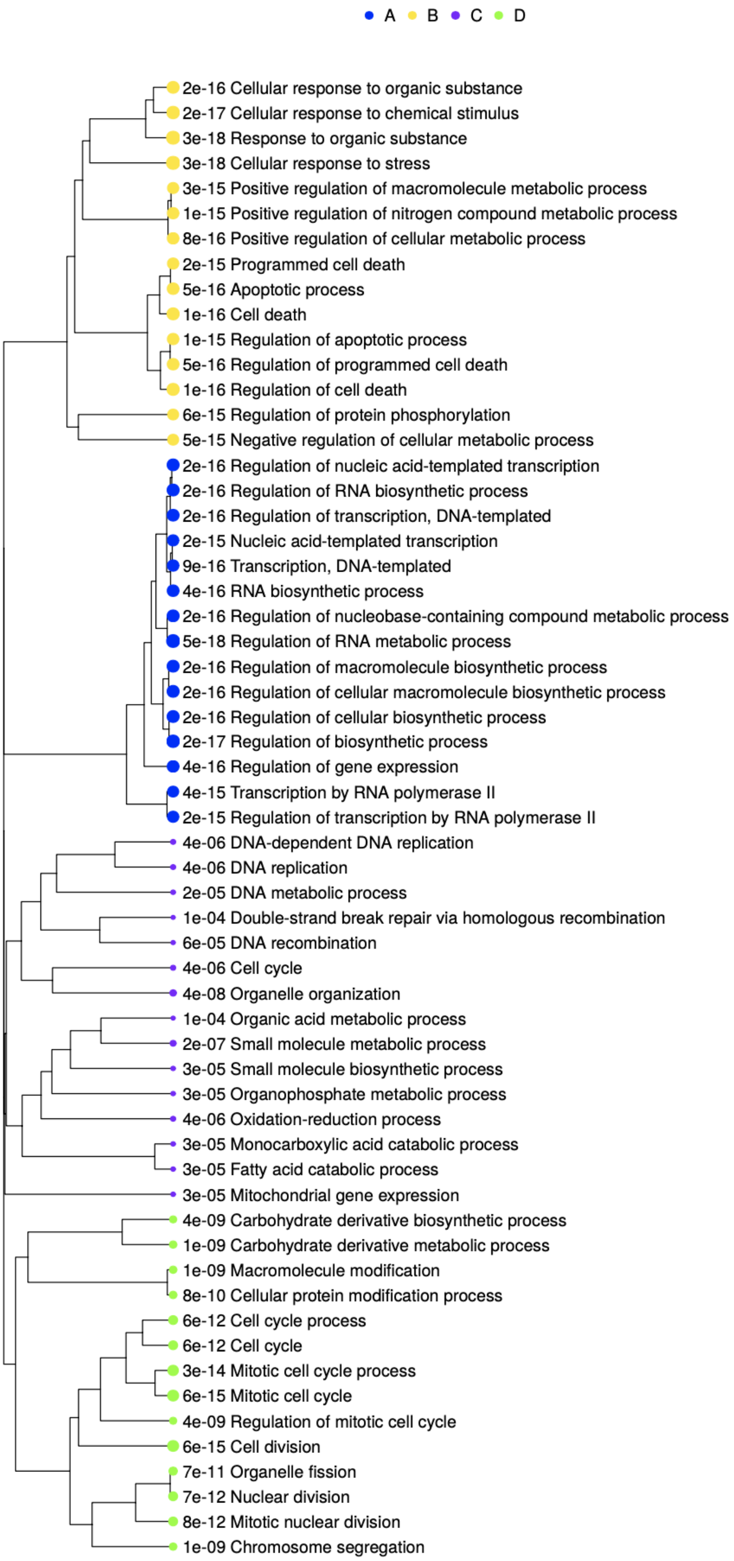

Supplemental Figure 4B-C. CCF642-34 is selective for PDIA1 inhibition-induced ER stress response pathway. Gene set enrichment analysis of differential expression A) Hierarchical clustering and heatmap of the gene expression in MM1.S cells treated with vehicle, CCF642-34, or CCF642, all genes were ranked by standard deviation across all samples and the top 5000 significantly expressed genes were used for unbiased hierarchical clustering using web-based server http://bioinformatics.sdstate.edu/idep/. B) Gene set enrichment analysis of expression data from MM1.S cells treated with PDI inhibitors. Genes are ranked by standard deviation among top 5000 significantly expressed genes. C) Color-coded gene sets in B are shown in a tree format. All data analysis was performed using web-based server http://bioinformatics.sdstate.edu/idep/.

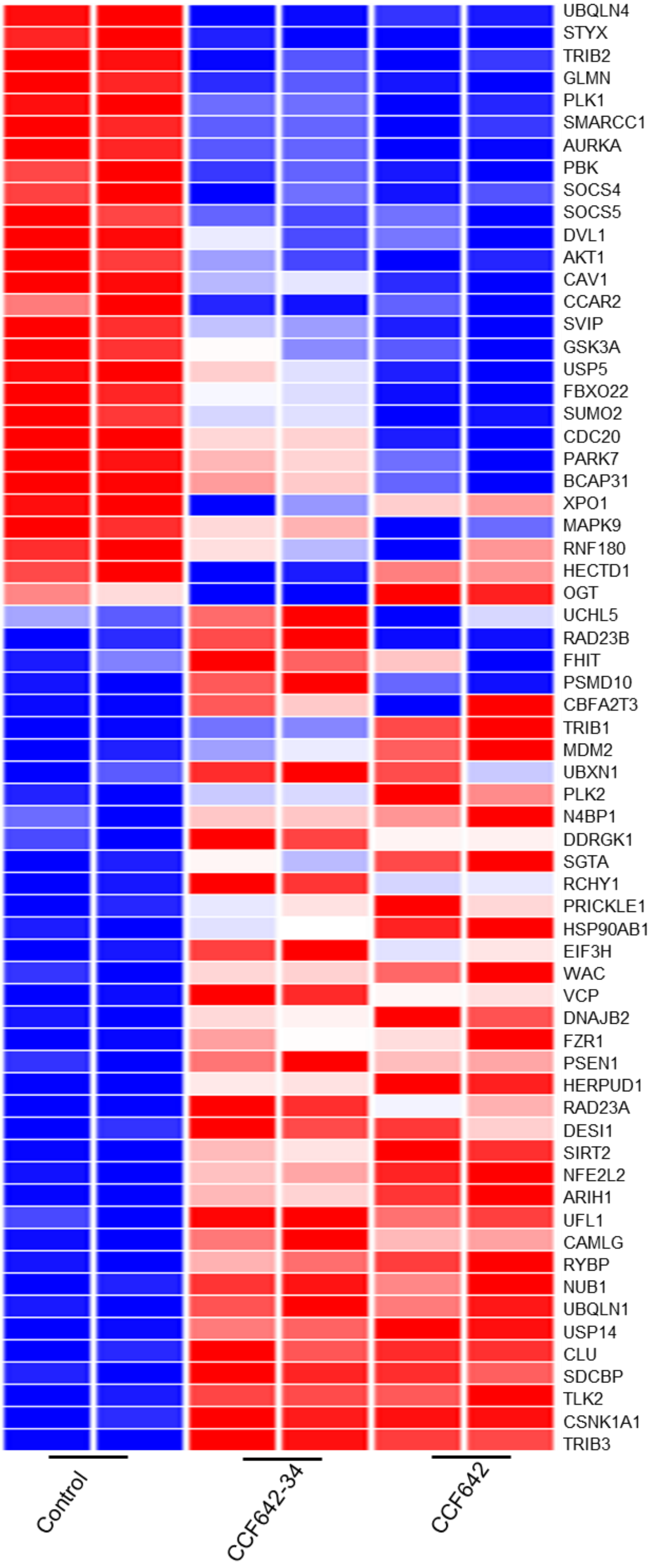

Supplemental Figure 4D. **Ubiquitin catabolism comparison after CCF642 and CCF642-34 treatment.** After treatment with CCF642 and CCF642-34, genes that are related to ubiquitin catabolism mediated by proteasome were compared in a heatmap.

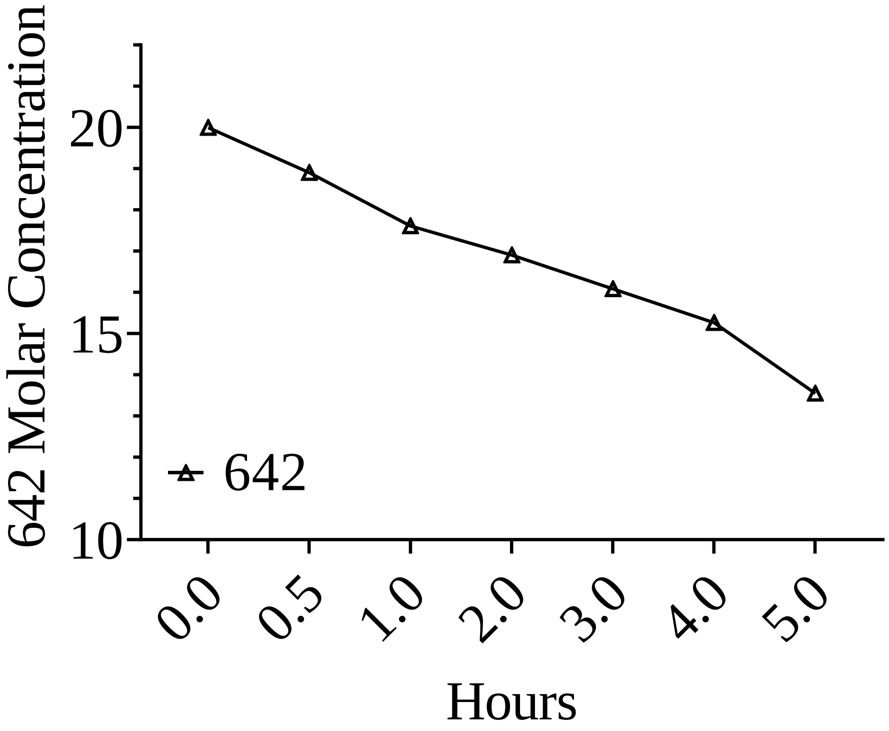

Supplemental Figure 5. The stability of CCF642 in human liver microsomes. The 20 μM CCF642 were incubated with 0.25mg/mL of human liver microsomes for indicated time. The residual compounds were measured by HPLC interfaced with reverse phase C18 column using 280 nm and 245 nm detection wavelength. The standard curve of the area under the peak at two wavelength were used to estimate the remaining compound. Data is representative of two independent set of experiments
