# Supplementary material for: Therapeutic Targeting of Protein Disulfide Isomerase PDIA1 in Multiple Myeloma
Source: Cancers (Basel). 2021 May 28;13(11):2649. doi: 10.3390/cancers13112649 (PMC8198550; doi:10.3390/cancers13112649)

Figure 4. Uncropped blots.

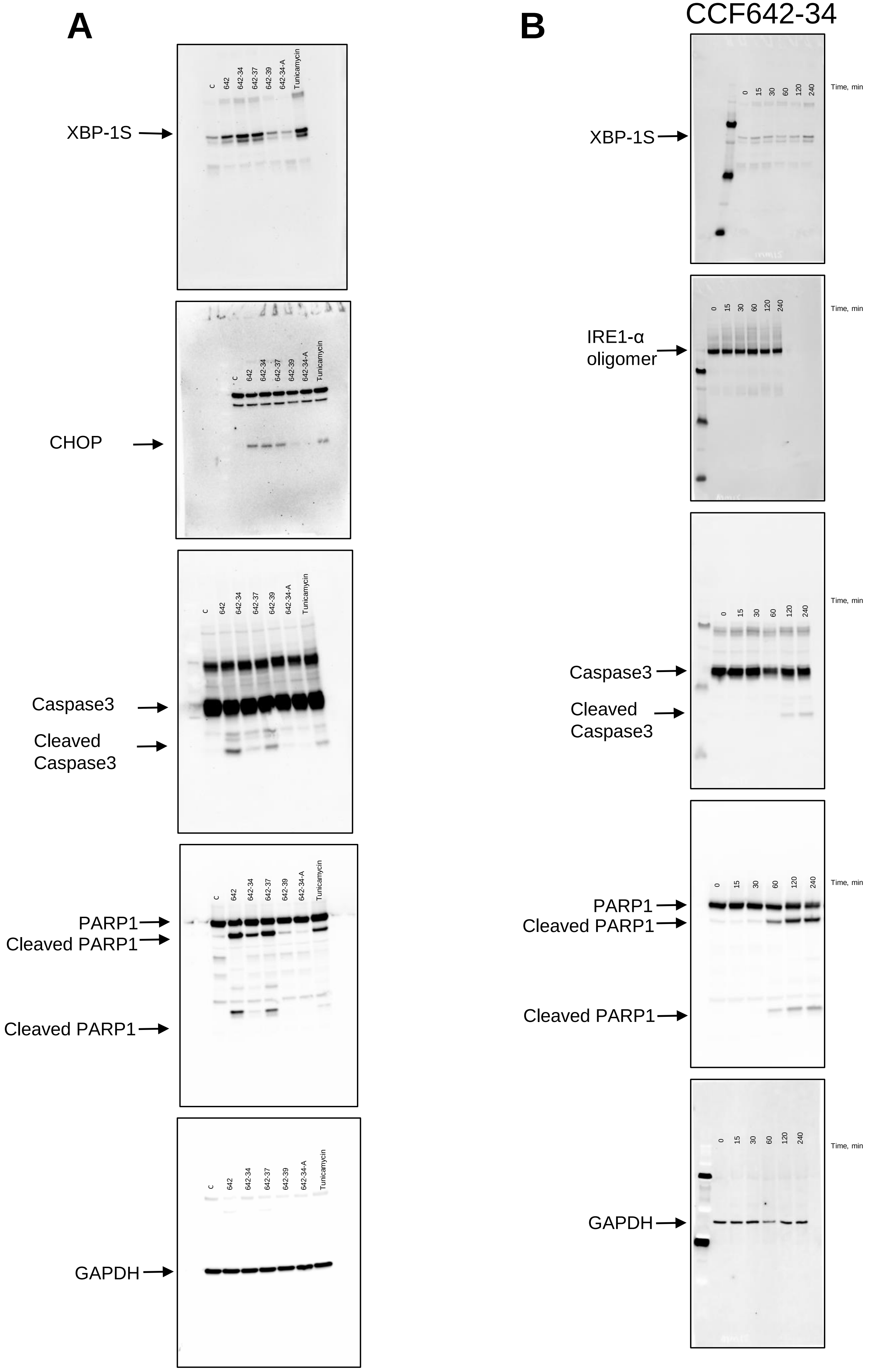

Supplemental Figure 2D. Uncropped blots.

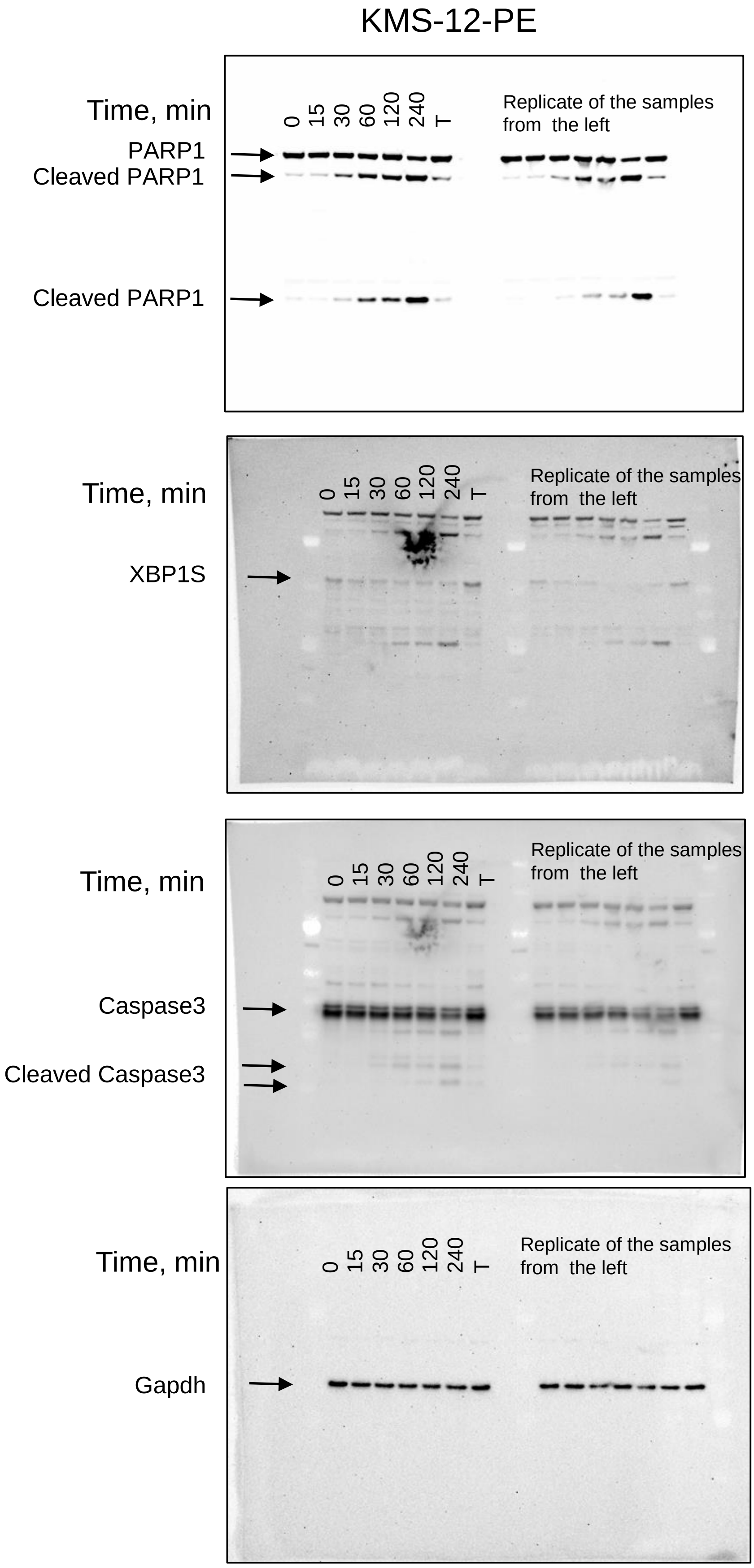

Supplemental Figure 2D. Uncropped blots.

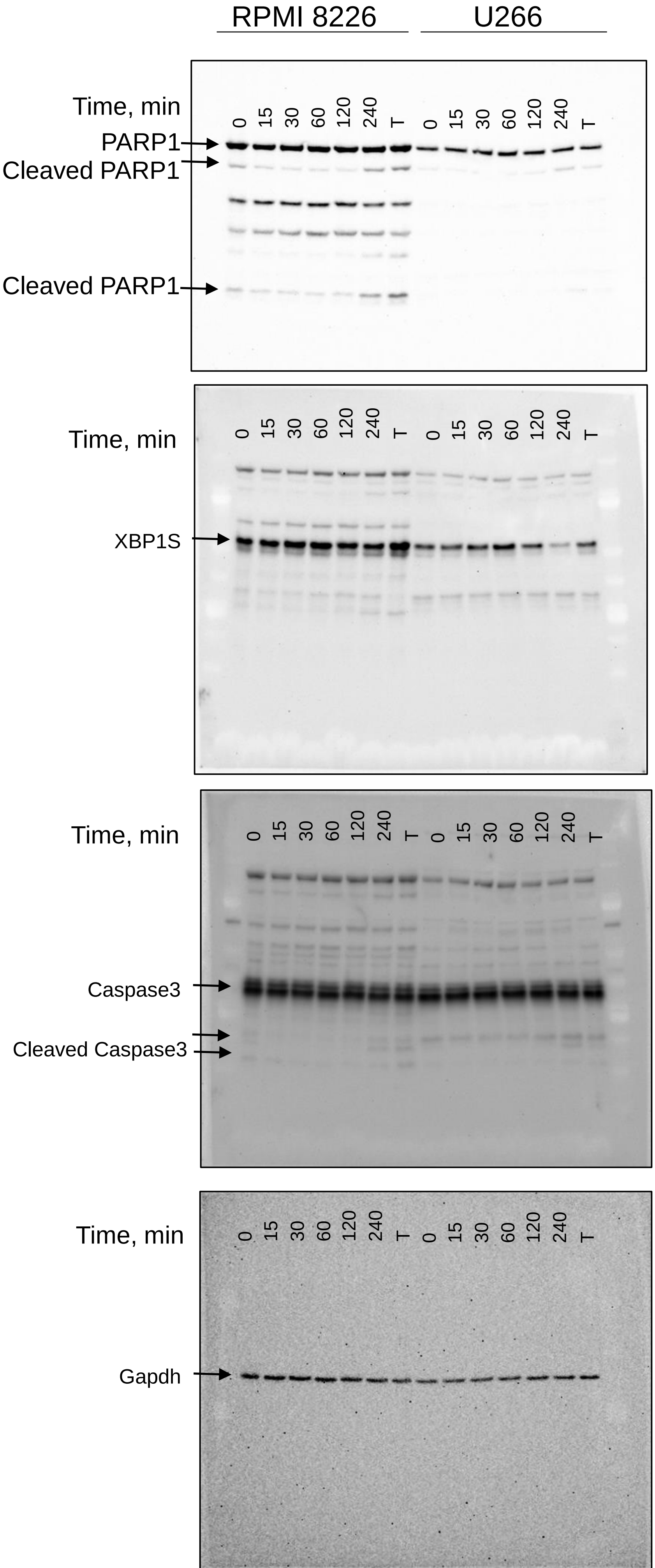

Supplement: Supplementary file 1 [file cancers-13-02649-s001.zip › Cancers_supp_file/File S1.pdf]
